# Psychometric evaluation of a newly developed measure of emotionalism after stroke (TEARS-Q)

**DOI:** 10.1177/0269215520981727

**Published:** 2020-12-21

**Authors:** Niall M Broomfield, Robert West, Allan House, Theresa Munyombwe, Mark Barber, Fergus Gracey, David C Gillespie, Matthew Walters

**Affiliations:** 1 Department of Clinical Psychology and Psychological Therapies, Norwich Medical School, University of East Anglia, Norwich, Norfolk, UK; 2 Institute of Cardiovascular and Medical Sciences, University of Glasgow, Glasgow, UK; 3 Leeds Institute of Health Sciences, University of Leeds, Leeds, West Yorkshire, UK; 4 University Hospital Monklands, Airdrie, UK; 5 Glasgow Caledonian University, Glasgow, UK; 6 Department of Clinical Neurosciences, Royal Infirmary of Edinburgh, Edinburgh, UK

**Keywords:** Stroke, emotionalism, mental health

## Abstract

**Objective:**

To evaluate, psychometrically, a new measure of tearful emotionalism following stroke: Testing Emotionalism After Recent Stroke – Questionnaire (TEARS-Q).

**Setting:**

Acute stroke units based in nine Scottish hospitals, in the context of a longitudinal cohort study of post-stroke emotionalism.

**Subjects:**

A total of 224 clinically diagnosed stroke survivors recruited between October 1st 2015 and September 30th 2018, within 2 weeks of their stroke.

**Measures:**

The measure was the self-report questionnaire TEARS-Q, constructed based on post-stroke tearful emotionalism diagnostic criteria: (i) increased tearfulness, (ii) crying comes on suddenly, with no warning (iii) crying not under usual social control and (iv) crying episodes occur at least once weekly. The reference standard was presence/absence of emotionalism on a diagnostic, semi-structured post-stroke emotionalism interview, administered at the same assessment point. Stroke, mood, cognition and functional outcome measures were also completed by the subjects.

**Results:**

A total of 97 subjects were female, with a mean age 65.1 years. 205 subjects had sustained ischaemic stroke. 61 subjects were classified as mild stroke. TEARS-Q was internally consistent (Cronbach’s alpha 0.87). TEARS-Q scores readily discriminated the two groups, with a mean difference of −7.18, 95% CI (−8.07 to −6.29). A cut off score of 2 on TEARS-Q correctly identified 53 of the 61 stroke survivors with tearful emotionalism and 140 of the 156 stroke survivors without tearful emotionalism. One factor accounted for 57% of the item response variance, and all eight TEARS-Q items acceptably discriminated underlying emotionalism.

**Conclusion:**

TEARS-Q accurately diagnoses tearful emotionalism after stroke.

## Introduction

A recent Cochrane review highlighted the need for a psychometrically robust, standardised measure of post-stroke emotionalism. A reliable, valid scale will ensure post-stroke emotionalism is correctly detected and not confused with depression.^1^

Post Stroke Emotionalism is characterised by lessening of ability to control emotional expression, in particular unheralded crying episodes not under personal control.^2,3^ Developing a robust psychometric measure of emotionalism following stroke is an important development for stroke care. Post-stroke emotionalism is common, affecting 20% of stroke survivors at the acute/post-acute phase and 12% at 1 year.^4^ Post-stroke emotionalism can impact engagement with physical rehabilitation, causing patients to avoid therapies due to embarrassment and fear of uncontrolled emotional episodes.^5^ Post-stroke emotionalism can also cause avoidance of many other face-to-face social interactions.^1^

Two non-stroke specific emotionalism measures have been previously developed: the Pathological Laughter and Crying Scale,^6^ and Centre for Neurological Sciences –Lability Scale.^7^ Both measures have limitations and undetermined psychometric properties in stroke. Neither was derived based on consensus diagnostic criteria or a clinical formulation of post-stroke emotionalism that separates laughter and crying components. Neither has cut-off scores to determine emotionalism caseness. Both measures blend evaluation of uncontrolled crying and uncontrolled laughter.^4^ With no derived sub-scale scores for separate components, the assessment becomes muddled, rendering the total score difficult to interpret clinically.

We therefore sought to evaluate the psychometric properties of a new post-stroke tearful emotionalism (crying) measure – Testing Emotionalism After Recent Stroke-Questionnaire (TEARS-Q).

## Method

Recruitment took place between October 2015 and September 2018. The Testing Emotionalism After Recent Stroke cohort study (Testing Emotionalism After Recent Stroke: NHS Research Scotland Stroke Research Network Identification 18980; https://www.stroke.org.uk/research/understanding-difficulty-controlling-emotions-after-stroke; full protocol from first author) was approved a priori by Scotland A Research Ethics Committee (Integrated Research Application System reference 157483). The study was funded by The Stroke Association, United Kingdom. NHS Greater Glasgow and Clyde Research and Development sponsored the study and held responsibility for its integrity and conduct.

### Scale development

Testing Emotionalism After Recent Stroke – Questionnaire (TEARS-Q) was developed following an initial literature review, content analysis of accounts of post-stroke emotionalism, and appraisal of the form and content of existing emotionalism measures. These initial steps: (i) clarified the nature of the target construct of post-stroke emotionalism, and (ii) identified problems with existing measures. The clinical formulation of post-stroke emotionalism comprising separate crying and laughter components was discussed by the study authors and it was agreed that a crying-only measure was a more necessary scale for clinical practice.

We structured the new measure based on published, widely accepted post-stroke emotionalism crying diagnostic criteria^2,3,8,9^: (i) increased tearfulness, (ii) crying comes on suddenly, with no warning (iii) crying not under usual social control and (iv) crying episodes occur at least once weekly. Two items were drafted to address each diagnostic criterion:

[i]: ‘I feel more tearful in the past 2 weeks than before the stroke’; ‘I have actually cried more in the past 2 weeks than before the stroke’.
[ii]: ‘My crying comes on suddenly, with only a few seconds or no warning’; ‘My crying comes on when I am not expecting it’.
[iii]: ‘When my crying comes on, I cannot control or stop it’; ‘I cry in situations I would not have cried in before the stroke’.
[iv] One item addressed the frequency criterion ‘I cry in this way at least once per week or more often’.

A final item was added to distinguish post-stroke emotionalism from depressed mood: ‘My crying comes on even if I do not feel sad at the time’.

All items were valenced identically and a five-point Likert scale was added to record respondent agreement: ‘Strongly Agree, Agree, Unsure, Disagree, Strongly Disagree’, scored 2,1,0,0,0 to detect positive evidence of symptoms. An instructional set was developed orienting respondents to focus on changes to specifically crying, since their stroke. The reference ‘*in the past 2* *weeks*’ provided a stable assessment timeframe within which to compare, making TEARS-Q a present state not trait measure, suitable for monitoring change over time. To allow for rapid (two item) post-stroke emotionalism clinical screening, a discontinue rule was integrated whereby ‘disagree’ or ‘strongly disagree’ responses on TEARS-Q-Q Item 1 and Item 2 lead to discontinuation of the measure (see Supplemental Appendix 1). The final TEARS-Q measure was endorsed by four expert study contributors and a person with personal experience of stroke.

### Psychometric evaluation

Study participants were recruited October 1st 2015 to September 30th 2018 within 2 weeks of stroke from acute stroke units in the context of a longitudinal post-stroke emotionalism cohort study across nine Scottish hospitals. All participants gave written informed consent. All participants were male or non-pregnant female, ⩾18 years of age, with clinical diagnosis of ischaemic or haemorrhagic stroke. Individuals with aphasia on Frenchay Aphasia Screening Test^10^ were excluded. Individuals with subarachnoid haemorrhage, other extra-axial bleeds or Transient Ischaemic Attack were excluded. Individuals with severe concurrent medical conditions or with life expectancy ⩽3 months were excluded, as were individuals lacking spoken English or with severe distressing behaviours secondary to stroke or dementia which in the opinion of the referring clinical team precluded participation. All assessments were conducted on the hospital wards within 2 weeks of the stroke event. Findings are based on baseline data.

The reference standard was Testing Emotionalism After Recent Stroke- Diagnostic Interview (see Supplemental Material Information, copy also available from first author). This was developed and adopted in the absence of any published, standardised schedule. The emotionalism diagnostic interview is detailed, semi-structured and comprises sections on post-stroke crying- screen questions, case characteristics, frequency and impact; post-stroke laughter- screen questions, case characteristics; diagnostic summary. Stroke research nurses, pre-trained by NB and AH, administered Testing Emotionalism After Recent Stroke – Diagnostic Interview. Other interview-based assessments were the Abbreviated Mental Test,^11^ Barthel Activities of Daily Living Index^12^ and National Institute of Health Stroke Scale.^13^

The index test was TEARS-Q questionnaire (see Supplemental Appendix 1). Help to complete was offered to a minority of participants, mainly in the form of reading out the questions verbatim, with about 10% of the total sample having the item put to them in words additional to the verbatim text. Participants also completed the Hospital Anxiety and Depression Scale,^14^ EuroQol Five Dimensions General Health Status Scale^15^ and Scottish Government Index of Multiple Deprivation.^16^

To compare study participants with and without diagnosed emotionalism, continuous data were summarised using means and standard deviations, categorical data by frequencies and percentages. Cross-tabulation was provided by diagnostic interview definition of caseness with indicative tests provided by either Student’s *t*-tests for continuous variables or Pearson’s Chi-square tests for categorical variables.

The TEARS-Q measure was formulated so that the question responses are scored by assigning 2 to the ‘strongly agree’ category, 1 to the ‘agree’ category and 0 to the other 3 categories: that is scored 2,1,0,0,0. The scores for the eight questions are then summed to provide the TEARS-Q measure total score, ranging from 0 to 16.

To check for dimensionality of TEARS-Q, Exploratory Factor Analysis was conducted.^17^ We examined the scree plot, eigenvalues, and magnitudes of item loadings. Establishing uni-dimensionality would mean that the TEARS-Q measure was dominated by aspects of emotionalism.

The TEARS-Q measure might have potential to be used as a diagnostic test, given a suitable cut point. To explore this possibility, a Receiver-Operator Characteristic curve was constructed. This relates the sensitivity and specificity of the TEARS-Q measure for a range of potential cut points. Sensitivity is the proportion of positive cases according to the diagnostic interview that are correctly identified using the TEARS-Q measure. Specificity is the proportion of negatives cases according to the diagnostic interview that are correctly identified using the TEARS-Q measure. An optimal cut point was selected by maximising the sum of sensitivity and specificity, also known as the Youden cut point. Having established this cut point, a 2x2 table was constructed to show the performance of the measure and from which positive and negative predictive values were derived. The positive predictive value of TEARS-Q is the probability that participants who screen positive (above the cut-point) on the measure truly have emotionalism. The negative predictive value of TEARS-Q is the probability that participants who screen negative (below the cut-point) truly do not have emotionalism.

During the administration of the TEARS-Q questionnaire, the first two questions (Item 1 and Item 2; see Supplemental Appendix 1) were utilised as stop/continue criteria, to minimise participant burden. This two-item measure might be considered as a quick tool for establishing patients who are likely to have emotionalism, therefore sensitivity and specificity are also reported for this.

For reference standard check, the first author, an experienced stroke clinical psychologist, assessed 32 participants blind to Testing Emotionalism After Recent Stroke-Diagnostic Interview outcome.

## Results

### Participants

There were 277 participants in the Testing Emotionalism After Recent Stroke cohort study, and 224 of these provided data within 2 weeks of their stroke for Testing Emotionalism After Recent Stroke-Questionnaire (TEARS-Q) as well as a diagnostic interview. Characteristics of the sample of 224 participants are summarised in Table 1.

**Table 1.** Characteristics for participants with and without post-stroke emotionalism.

Characteristic	Levels	No diagnosis of post-stroke emotionalism	Diagnosis of post-stroke emotionalism	Total cohort	*P*
Number of participants		162	62	224	
Age when stroke occurs	Mean (SD)	67.0 (14.4)	59.7 (14.0)	65.0 (14.6)	0.001
Sex	Female	64 (39.5%)	33 (53.2%)	97 (43.3%)	0.088
	Male	98 (60.5%)	29 (46.8%)	127 (56.7%)	
Scottish index of multiple deprivation rank	Mean (SD)	2789 (2077)	2560 (1935)	2726 (2037)	0.455
Education	Primary	3 (1.9)	2 (3.2)	5 (2.2)	0.421
	Secondary	99 (61.1)	45 (72.6)	144 (64.3)	
	University	28 (17.3)	6 (9.7)	34 (15.2)	
	Other	24 (14.8)	6 (9.7)	30 (13.4)	
Any comorbidities	No	119 (73.5)	43 (69.4)	162 (72.3)	0.653
	Yes	42 (25.9)	19 (30.6)	61 (27.2)	
	Unknown	1 (0.6)		1 (0.4)	
EQ5D5L^a^ mobility	Mean (SD)	2.3 (1.2)	2.4 (1.3)	2.3 (1.3)	0.547
EQ5D5L^a^ self care	Mean (SD)	1.8 (1.0)	2.1 (1.2)	1.9 (1.1)	0.098
EQ5D5L^a^ usual activities	Mean (SD)	2.5 (1.3)	2.9 (1.4)	2.6 (1.3)	0.021
EQ5D5L^a^ pain/discomfort	Mean (SD)	1.8 (1.0)	2.0 (1.3)	1.8 (1.1)	0.193
EQ5D5L^a^ anxiety/depression	Mean (SD)	1.4 (0.7)	2.2 (1.2)	1.6 (1.0)	<0.001
EQ5D5L^a^ health today	Mean (SD)	66.4 (19.4)	58.4 (21.1)	64.2 (20.1)	0.008
Barthel activities of daily living index	Mean (SD)	16.7 (4.4)	16.1 (5.0)	16.5 (4.6)	0.342
Abbreviated mental test	Mean (SD)	7.9 (1.0)	7.6 (1.1)	7.8 (1.0)	0.08
HADS^b^ depression	Mean (SD)	3.9 (3.7)	6.3 (4.3)	4.5 (4.0)	<0.001
HADS^b^ anxiety	Mean (SD)	4.6 (3.9)	8.3 (5.4)	5.6 (4.6)	<0.001
NIHSS^c^	Mean (SD)	4.4 (4.6)	5.4 (5.2)	4.7 (4.8)	0.406
	(0, 5)	44 (78.6)	17 (70.8)	61 (76.2)	0.356
	(6, 16)	11 (19.6)	5 (20.8)	16 (20.0)	
	(17, 23)	1 (1.8)	2 (8.3)	3 (3.8)	
Stroke type	Infarct	146 (90.7)	59 (95.2)	205 (91.9)	0.409
	Haemorrhage	15 (9.3)	3 (4.8)	18 (8.1)	
Oxford classification of stroke	Total anterior circulation stroke	8 (5.1)	2 (3.4)	10 (4.6)	0.524
	Partial anterior circulation stroke	57 (36.3)	17 (28.8)	74 (34.3)	
	Lacunar stroke	53 (33.8)	26 (44.1)	79 (36.6)	
	Posterior circulation stroke	39 (24.8)	14 (23.7)	53 (24.5)	

a EQ5D5L: EuroQol Five Dimensions General Health Status Scale.

bH ADS: Hospital Anxiety and Depression Scale.

c NIHSS: National Institutes of Health Stroke Scale.

### Item responses

Table 2 provides the response frequencies for the questions for the Testing Emotionalism After Recent Stroke – Questionnaire (TEARS-Q) measure. Note that only the first two questions are administered if an individual answers ‘disagree’ or ‘strongly’ disagree’ on either item.

**Table 2.** Item content and response frequencies for testing emotionalism after recent stroke – questionnaire.

Question	Score assigned		
	0 (Unsure/disagree/strongly disagree)	1 (Agree)	2 (Strongly agree)
Q1. I feel more tearful in the past 2 weeks than before the stroke	147 (65.6%)	46 (20.5%)	31 (13.8%)
Q2. I have actually cried more in the past 2 weeks than before the stroke	163 (72.8%)	32 (14.3%)	29 (13.0%)
Q3. My crying comes on suddenly, with only a few seconds or no warning	16 (20.8%)	40 (52.0%)	21 (27.3%)
Q4. My crying comes on when I am not expecting it	30 (39.0%)	31 (40.3%)	16 (20.8%)
Q5. When my crying comes on, I cannot control or stop it	34 (44.1%)	29 (37.7%)	14 (18.2%)
Q6. I cry in situations I would not have cried in before the stroke	37 (48.1%)	25 (32.5%)	15 (19.5%)
Q7. I cry in this way at least once per week or more often	25 (32.5%)	40 (52.0%)	12 (15.6%)
Q8. My crying comes on even if I do not feel sad at the time	24 (31.2%)	38 (49.4%)	15 (19.5%)

### Internal consistency

To assess how reliably TEARS-Q measures what it should measure, internal consistency data were computed. Using the post-stroke emotionalism diagnosed group, Cronbach’s alpha was 0.87, item-deletion alphas were high, and there was minimal variation between values (mean alpha = 0.87, range 0.84–0.87). Mean corrected item-total correlation was 0.68. These investigations show TEARS-Q is a reliable measure of emotionalism.

### Discriminant validity

The total TEARS-Q measure discriminates well between those with and those without emotionalism, as determined by interview. The mean total TEARS-Q score for participants with post-stroke emotionalism was 7.8 (standard deviation 4.8) range 0 to 16. For participants without post-stroke emotionalism, the mean was 0.6 (standard deviation 1.9), range 0–11. Neither age nor sex significantly impacted TEARS-Q score whilst emotionalism added on average 7.2 points (95% CI [6.3, 8.1]).

### Diagnostic accuracy

**Figure 1.**
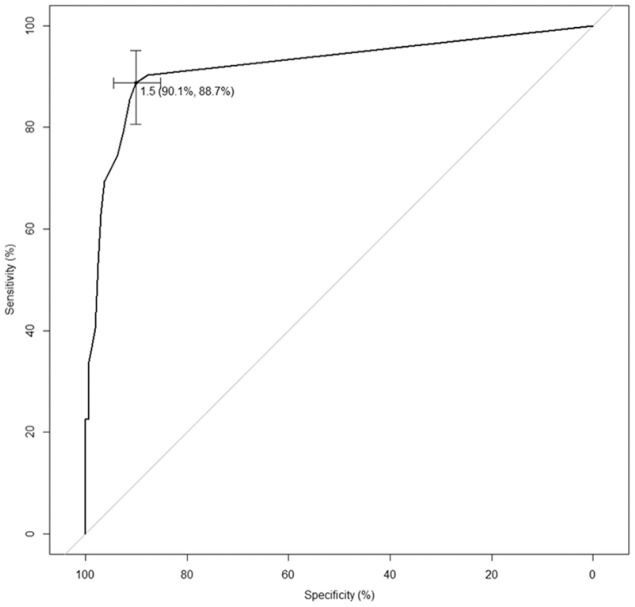
Receiver operating characteristic curve for testing emotionalism after recent stroke – questionnaire. This gives a graphical representation of the values of sensitivity and specificity for each potential cut point for the TEARS-Q measure. The Youden cut point maximises the sum of sensitivity and specificity and occurs at 1.5 yielding a specificity of 90.1% and a sensitivity of 88.7%.

The Receiver-Operator Characteristic curve for the TEARS-Q total score can be seen in Figure 1. This also shows the cut point for which the sum of the sensitivity and specificity is maximised: that is 1.5. The cross at the cut point gives the sensitivity and specificity calculated using 1.5 as the cut point and shows graphically the 95% confidence intervals for sensitivity and specificity. The area under the Receiver–Operator Characteristic curve is 0.929 indicating outstanding performance of the TEARS-Q measure.^18^

Since TEARS-Q scores have to be whole numbers, for clinical purposes the best cut-point is 2. In other words, TEARS-Q best predicts post-stroke emotionalism for those patients with a total TEARS-Q score of 2 or more, and for patients who score 0 or 1, it predicts no emotionalism. Data on this relationship between diagnosis based on interview and diagnosis based on the TEARS-Q measure are shown in Table 3. There is also information underneath Table 3 on TEARS-Q measure sensitivity, the proportion of positive cases according to the diagnostic interview that are correctly identified using the TEARS-Q measure and TEARS-Q specificity, the proportion of negatives cases according to the diagnostic interview that are correctly identified using the TEARS-Q measure. High specificity of 90% makes TEARS-Q an excellent instrument for ruling in emotionalism.

**Table 3.** The relationship between diagnosis based on interview and diagnosis, based on eight-item TEARS-Q measure.

Number of patients	TEARS-Q	
	2 or more	0 or 1
Interview findings		
Post-stroke emotionalism present	53	8
No post-stroke emotionalism	16	140

Based on this table, a score of 2 or more on TEARS-Q predicts post-stroke emotionalism and score of 0 or 1 predicts absence of emotionalism. The sensitivity of the TEARS-Q test is 53/61, which is 87% and the specificity is 140/156, which is 90%. The positive predictive value is 53/69, which is 77% and the negative predictive value is 140/148, which is 95%.

### Dimensionality

**Figure 2.**
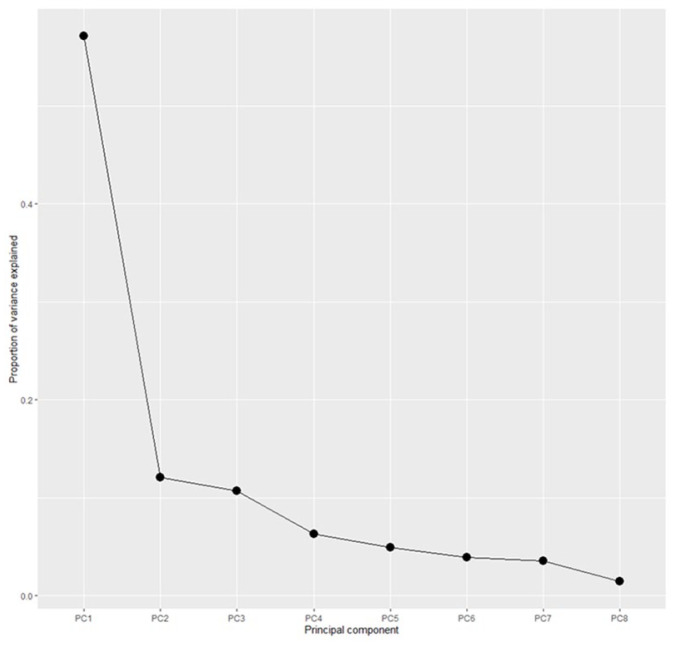
Scree plot for TEARS-Q indicating one dimension. The pronounced ‘elbow’ shows that most of the information comes from the first component of a principal component analysis: that is TEARS-Q is a unidimensional measure.

A principal component analysis of the eight item TEARS-Q responses reveals that the first component represents 57.1% of the variation. As is evident in Figure 2, the scree plot includes a pronounced ‘elbow’ which shows that most of the information comes from the first component of the principal component analysis. The item loadings for the first principal component vary from 0.255 to 0.403 showing an almost even weighting of the questions. It is clear from these investigations that the TEARS-Q measure is a unidimensional measure, in other words it measures the single concept of emotionalism.

### Two-item screen

The first two questions of TEARS-Q were used as a screening tool. If a participant in the study answered either ‘disagree’ or ‘strongly disagree’ to both of these, then the further six questions were not asked. Although the main purpose of this was to avoid burden on the participant who had recently suffered a stroke, this in effect provides a two-item screening tool. Table 4 tabulates the stop or continue rule with the diagnosis of post-stroke emotionalism. As a stand-alone screening tool, used to identify post-stroke emotionalism without the further six TEARS-Q questions, the sensitivity, the proportion of participants with emotionalism that are flagged as ‘continue’ by the two-item screen, is 56/62 which is 90.3%. The specificity, the proportion without emotionalism flagged as ‘stop’, is 133/162 which is 82.1%.

**Table 4.** The relationship between diagnosis based on interview and diagnosis, based on two-item TEARS-Q measure.

Number of patients	TEARS-Q two-item screen	
	Continue	Stop
Interview findings		
True post-stroke emotionalism	56	6
No post-stroke emotionalism	29	133

Based on this table, as a stand-alone screening tool used to identify post-stroke emotionalism without the further six TEARS-Q questions, the sensitivity, the proportion of participants with emotionalism that are flagged as ‘continue’ by the two-item screen is 90.3%. The specificity, the proportion without emotionalism flagged as ‘stop’ is 82.1%.

### Reference standard check

As a check on the reference standard, the first author assessed 32 participants (14% of whole sample) blind to Testing Emotionalism After Recent Stroke-Diagnostic Interview outcome. There was agreement on 31 of 32 cases.

## Discussion

Data from this validation study suggest acceptable psychometric properties, for clinical, research and trialist usage, of Testing Emotionalism After Recent Stroke – Questionnaire (TEARS-Q). TEARS-Q is internally consistent, and people diagnosed with post-stroke emotionalism score 7.2 points above people without post-stroke emotionalism. TEARS-Q is sensitive and specific to detect tearful emotionalism. A cut off score of 2 or more correctly identifies 87% of stroke survivors with post-stroke emotionalism and 90% of stroke survivors without post-stroke emotionalism. TEARS-Q is a unidimensional measure, dominated by emotionalism without contamination from other conditions such as depression and anxiety. The area under the curve is high showing strong performance of this new measure.

One factor accounts for 57% of item response variance, and all eight items acceptably discriminate the underlying latent post-stroke emotionalism variable, with item ‘my crying comes on when I am not expecting it’ offering the greatest discrimination and reliability. This is intuitive conceptually, given post-stroke emotionalism core clinical features: unexpected tearfulness, sudden and unheralded, not reflecting inner sadness.^2,8,9^

This brief, psychometrically robust screening measure improves our ability to accurately detect, diagnose and treat tearful emotionalism and might significantly improve stroke care. Collection of routine, consecutive series TEARS-Q data in a large stroke centre is needed however, as is further validation work ensuring independent administration, and to develop parallel informant and post-stroke emotionalism laughter measures.

Testing Emotionalism After Recent Stroke – Questionnaire (TEARS-Q) is the first self-report questionnaire developed for tearful emotionalism in stroke. The establishment of acceptable psychometrics for TEARS-Q eliminates the need for reliance in stroke settings on previous emotionalism measures, not developed to assess tearful emotionalism in stroke populations.^6,7^ That said, although we are concerned here with the questionnaire’s performance after stroke, there is no *a priori* reason to consider that the phenomenon of emotionalism is fundamentally different in other conditions it which it is commonly encountered – such as multiple sclerosis, amyotrophic lateral sclerosis and traumatic brain injury.^19^ Our findings therefore have a potentially wider application in neuro-rehabilitation research and practice.

Study limitations and biases must be considered. The majority of participants were West of Scotland resident so replication using a broader UK population and across other countries and cultures is needed. The sample was relatively young with a bias towards mild stroke meaning that the measure was not validated in the whole stroke severity range, and participants with aphasia did not complete the assessments.

Whilst the original measure item choice and wording were guided by diagnostic criteria for tearful emotionalism and expert consensus, stroke nursing, occupational therapy, speech and language and physiotherapy professions were not consulted. Moreover, final items were not co-produced by a stroke survivor or sense-checked by a stroke survivor specifically with post-stroke emotionalism.

We developed the diagnostic interview reference standard because there was no published, standardised interview diagnostic schedule to adopt. An alternate reference standard could have been post-stroke emotionalism clinical diagnosis from an experienced stroke clinician, with the disadvantage that it would not have been standardised. Clinical information and reference standard results were not available to participants completing the measure however clinical information was available to the nurses who delivered the measure and reference standard, and the time interval between the measure and reference standard delivery was minimal (same day). Moreover, since positive and negative predictive values change with prevalence, data on these parameters only apply to the acute stroke phase. Nonetheless, the data suggest TEARS-Q performs accurately in clinical settings, comparing to a semi-structured, diagnostic post-stroke emotionalism interview. Further validation work will need independent administration of TEARS-Q and the reference standard.

The 2 week scale time frame of TEARS-Q is potentially restrictive and as TEARS-Q is self-report, some stroke survivors with cognitive or communication difficulties will be unable to complete it. We are developing an informant version, TEARS-Questionnaire Informant and observational and aphasia friendly versions will be needed for people with stroke related language problems. TEARS-Q is brief however and to minimise measure burden, we integrated fewest possible items and a discontinue rule. Moreover, the five-point Likert scale with ‘unsure’ middle response may suit cognitive impaired and fatigued respondents, making it easier for assessors to read out scale descriptors.

TEARS-Q only assesses tearful emotionalism so a measure to detect the much rarer uncontrollable laughter is required. It could be argued that a single measure recording both would be desirable, as laughter and crying can occur in the same individual. However, this is rare with the crying only presentation far more common^4^ and given the much higher prevalence of crying, we felt a crying measure was the priority. As tearful emotionalism is not stroke specific, similar measures should be developed for other neurologic populations.

Our findings also have significant clinical implications for the stroke community including the fifth of stroke survivors who develop tearful emotionalism. TEARS-Q could become part of a standard assessment battery in hospital settings and there is potential for community use via family doctors. TEARS-Q is brief to complete and it also integrates a two-item screen. This has potential as a rapid clinical tool given the acceptable psychometric properties. The two-item screen is faster and puts less burden on the patient, but the specificity is markedly lower than the full TEARS-Q measure. High specificity is important to rule-in emotionalism.

Embedding TEARS-Q into stroke pathways will help service commissioners better understand post-stroke emotionalism and its impact and stroke trialists now have access to a standardised psychometric measure to quantify, monitor and evaluate outcomes. As the authors of the recent Cochrane review for post-stroke emotionalism interventions note: ‘We recommend that future trials investigating the effect of antidepressants in people with emotionalism after stroke should use a standardised method to diagnose emotionalism’.^1^

Most important, TEARS-Q improves the quality of psychological assessment and care for people living with stroke. Without such a measure, tearful emotionalism can be undiagnosed or misdiagnosed, and not treated appropriately. Post-stroke emotionalism is a syndrome of emotional expression not emotional experience but frequent crying episodes in the context of stroke adjustment can easily be interpreted as due to a depressive disorder. There is an association between post-stroke emotionalism and depression but not all people with post-stroke emotionalism are depressed.

Finally, what are the implications of our findings for further research? First, larger scale validation of TEARS-Q is needed – across the full range of stroke pathology and in other neurological disorders. Future psychometric research should seek to establish especially the test-retest reliability and sensitivity to change of the measure – of great importance for its use in treatment evaluations. There are a number of important questions that could be answered by research with a psychometrically robust measure – including the effect of early emotionalism on later outcomes, and particularly the effect on social participation caused by avoidance; factors associated with chronic or severe emotionalism, and of course response to pharmacological and non-pharmacological treatments.

**Clinical messages**

- Testing Emotionalism After Recent Stroke – Questionnaire (TEARS-Q) has acceptable psychometric properties to diagnose tearful emotionalism after stroke
- A score of zero or one suggests absent emotionalism, whereas a score of two or more out of 16 suggests emotionalism is present
- TEARS-Q also allows rapid (two item) screening of emotionalism, with good sensitivity but lower specificity than the full eight-item measure

## Supplemental Material

sj-pdf-1-cre-10.1177_0269215520981727 – Supplemental material for Psychometric evaluation of a newly developed measure of emotionalism after stroke (TEARS-Q) Click here for additional data file. Supplemental material, sj-pdf-1-cre-10.1177_0269215520981727 for Psychometric evaluation of a newly developed measure of emotionalism after stroke (TEARS-Q) by Niall M Broomfield, Robert West, Allan House, Theresa Munyombwe, Mark Barber, Fergus Gracey, David C Gillespie and Matthew Walters in Clinical Rehabilitation
