# Supplementary material for: Psychometric evaluation of a newly developed measure of emotionalism after stroke (TEARS-Q)
Source: Clin Rehabil. 2020 Dec 21;35(6):894–903. doi: 10.1177/0269215520981727 (PMC8191157; doi:10.1177/0269215520981727)
Supplement: sj-pdf-1-cre-10.1177_0269215520981727 – Supplemental material for Psychometric evaluation of a newly developed measure of emotionalism after stroke (TEARS-Q) [file sj-pdf-1-cre-10.1177_0269215520981727.pdf]

Appendix 1 Testing for Emotionalism After Recent Stroke-Questionnaire (TEARS-Q)

Stroke can cause changes to emotional expression (how we show our emotions), in particular through crying. The following statements relate to your pattern of crying since your stroke. Please indicate by circling one response how true each statement is for you, **in the past two weeks**.

1. I feel more tearful **in the past two weeks** than before the stroke

Strongly Agree Agree Unsure Disagree Strongly Disagree

2. I have actually cried more **in the past two weeks** than before the stroke

Strongly Agree Agree Unsure Disagree Strongly Disagree

*** If response is disagree or strongly disagree on both items 1 and 2, discontinue the test***

3. My crying comes on suddenly, with only a few seconds or no warning

Strongly Agree Agree Unsure Disagree Strongly Disagree

4. My crying comes on when I am not expecting it

Strongly Agree Agree Unsure Disagree Strongly Disagree

5. My crying comes on even if I do not feel sad at the time

Strongly Agree Agree Unsure Disagree Strongly Disagree

6. When my crying comes on, I cannot control or stop it

Strongly Agree Agree Unsure Disagree Strongly Disagree

7. I cry in situations I would not have cried in before the stroke

Strongly Agree Agree Unsure Disagree Strongly Disagree

8. I cry in this way at least once per week or more often

Strongly Agree Agree Unsure Disagree Strongly Disagree

**Scoring: strongly agree = 2, agree = 1, unsure = 0, disagree = 0, strongly disagree = 0
